# Case of a Woman at the Hague, on Whom the Section of the Symphysis of the Ossa Pubis Has Twice Been Performed with Success
*This article is extracted from the accounts of the two operations communicated by Professor Camper to the editors of a periodical Work, printed at Amsterdam, entitled, Algemeene Vaderlandsche Letteroeffeningen, and inserted in their volumes for the years 1784 and 1786.


**Published:** 1787

**Authors:** J. C. Damen

**Affiliations:** Surgeon at the Hague


### III. *Case* * *of a Woman at the Hague, on whom the Section of the Symphysis of the Ossa Pubis has twice been performed with Success, by Mr.* J. C. Damen, *Surgeon at the Hague.*

MRS. Cornelia Stols, the subject of this singular history, was, at the time the first of the two operations was performed, in her four and thirtieth year. She was free from any appearance of rickets, and in every respect well formed, except that her pelvis was thought to be somewhat too narrow. Its smallest diameter, which was from one os ischium to the other, was supposed to be about three inches; and its largest, from the ossa pubis to the os sacrum, about four.

In two former labours, in both of which she was attended by Mr. Damen, it had been deemed necessary to have recourse to the crotchet. In the second of these deliveries, he had been favoured with the assistance of three experienced practitioners of midwifery at the Hague, all of

* This article is extracted from the accounts of the two operations communicated by Professor Camper to the editors of a periodical Work, printed at Amsterdam, entitled, *Algemeene Vaderlandsche Letteroeffeningen*, and inserted in their volumes for the years 1784 and 1786.

whom whom agreed with him in opinion, that the pelvis was too narrow to allow the head of a full grown fœtus to paſs through it without being leſſened. This induced him, when the patient became pregnant a third time, to think of having recourſe to the ſection of the ſymphyſis; and in this idea he was confirmed by Profeſſor Camper, and Dr. Van de Laar, a phyſician man-midwife of great repute at the Hague, who having ſeverally examined the patient, and found a narrowneſs at the upper part of the lower pelvis, were of opinion, that the difficulty ariſing from this circumſtance might be obviated by the intended operation.

Mr. Damen was called to the patient on the 20th of October, 1783, about four in the afternoon. She had then ſtrong labour pains; and at eight o'clock the os uteri was fully dilated, and the head of the child felt reſting on the brim of the pelvis. The ſection of the ſymphyſis having been previouſly determined on, and the conſent of the patient and her friends obtained, nothing now ſeems to have been thought of but to fix on a favourable moment for performing it. Mr. Damen had prepared himſelf for this undertaking by the kind inſtructions of Profeſſor Camper, who, during his residence at the Hague, had carefully explained to him on a cadaver all the precautions necessary to be observed in it.

The operation was performed in the presence of Doctors Janssen, Van de Laar, and Hasselmann, soon after eight in the evening. The patient was placed in a proper situation on a couch, (the rectum and bladder having been previously emptied) and an incision made through the integuments, about the middle of the ossa pubis. The symphysis was easily divided, and the bones immediately separated so much, as to admit the finger of the operator very easily between them.

Mr. Damen was now able to introduce his hand into the uterus, and bringing down the feet of the child, delivered the patient of "a "healthy, strong boy, full grown, and of the "largest size." The placenta came away without any difficulty. After dressing the wound with dry lint, Mr. Damen applied a steel bandage, invented for these cases by Professor Camper *.

* A description of this bandage, by Professor Camper, is inserted in the *Nieuwe Vaderlandsche Letteroeffeningen*, Vol. V.

The urine came away involuntarily till the twelfth day after the operation, when the patient began to difcharge it again naturally. The wound was completely healed on the thirtieth day after the operation.

Profeffor Camper's bandage was at firft thought to be very ufeful, and perfectly anfwered the purpofe it is intended for in thefe cafes, that of keeping the bones of the pelvis together; but notwithftanding it was lined with fmooth leather, and well covered with flannel, the preffure it made on the hips was fo great, that on the thirtieth day it was found neceffary to lay it afide, and to fubftitute in its ftead a belt, feven inches broad, made of foft Turkey leather, lined with flannel, and provided with three buckles. When the wound was dreffed, it was neceffary to loofen only the undermoft buckle, fo that the offa pubis were kept always clofe together.

The patient was defired to lay conftantly on her back till the 28th of November, when fhe was allowed to ftand upright, and to walk a little for the firft time. She continued to avail herfelf of this permiffion every day more and more, and was foon able to perform her domeftic bufinefs as well as ever.

Dr. Fiſcher, profeſſor of phyſic and midwifery in the univerſity of Goettingen, who happened to be at the Hague in November 1783, and who viſited the patient with Mr. Damen, wrote the following account of her ſituation to Profeſſor Camper.

" I have ſeen to-day (November 28th) Mrs.

" Stols, and her child, with the utmoſt ſatis-

" faction, both of them in good health. The

" wound is almoſt healed, and ſhe can now

" ſtand upright, and walk without pain or dif-

" ficulty, as I have had an opportunity of ſee-

" ing, when ſhe made the trial for the firſt

" time."

On the 23d of June of the year following, when Profeſſor Camper was at the Hague, the patient was examined by that gentleman, Dr. Van de Laar, and Mr. Damen, and the following affidavit is given as the reſult of the examination, viz.

" Inquiry into the conſequences of the divi-

" ſion of the ſymphyſis pubis, performed on

" the 23d of October, 1783, by Mr. Damen,

" on the wife of Caſpar Stols, of the Hague.

" We the underwritten P. Camper, A. Van
" de Laar, and J. C. Damen, do certify, that
" we have examined, this 23d of June, 1784,

" at

" at the houſe of Mr. Damen, the wife of Mr. " Caſpar Stols, and have found that the oſſa pubis " are immovably united on the inſide of the pel- " vis, leaving a ſmall elevation all along that " union, which is not above the breadth of a " ſtraw; and which in women who have never " undergone ſuch an operation, is often much " more remarkable: and farther, that on the " outſide the connexion is alſo very evident; " but that a little below the middle of the oſſa " pubis, there is a place which is painful " when touched, and ſomewhat ſoft and ele- " vated. Dr. Camper ſuſpects a little matter " at this part, but which, he thinks, will eaſily " find its way outwardly, as hath already hap- " pened.—Mr. Damen thinks the left os pubis " is ſomewhat lower than the right: but this " difference has not been obſerved either by " Dr. Camper or Dr. Van de Laar.

" The urethra ſeems to be moveable on both " ſides inwardly, and to be not ſo attached to " the oſſa pubis as in other women.

" They find that the patient is ſometimes " unable to retain her urine when in an erect " poſture; but that ſhe is always free from this " inconvenience when in an horizontal poſi- " tion, and that oftentimes, for ſeveral days

" to-

"together, no urine is difcharged involunta-
"rily.

"All the other parts appear to be quite na-
"tural. She walks very fteadily, and came
"this evening, with her child on her arm, to
"the houfe of Mr. Damen.

"Some weeks after the operation, when fhe
"was walking and performing her domeftic
"bufinefs, a bubonocele, to which fhe had
"long been fubject, made its appearance again,
"but has fince been eafily kept up by a pro-
"per trufs.

"In all other refpects fhe appears to be in

"perfect health.

(Signed) "Petrus Camper.

"A. Van de Laar.

"J. C. Damen."

We come now to the account of the fecond operation, which was performed on the fame patient on the 11th of Auguft, 1785. The divifion of the fymphyfis was not fo eafily performed as before, and was followed by fymptoms which threatened the life of the patient, but which Mr. Damen fuppofed to be independent of the operation.

Mr. Damen was called to her at ten in the morning. The os uteri was then much dilated,

 but but the head of the child was fo high up, that he could not feel it by the touch. The fuccefs of the former operation, however, and the opinion he had formed of the impoffibility of a child's paffing alive through the pelvis of this patient, feems to have determined him to have immediate recourfe again to the fection of the fymphyfis pubis, without waiting the farther progrefs of the labour. He was confirmed in this refolution by Doctors Joriffen, Van de Laar, Haffelmann, Kafteele, and Huybert, who affifted him on this occafion; and who, having examined the pelvis, were of opinion, that it was not at all enlarged by the former operation, and that the fection was the only means by which the life of the child could be faved.

In cutting through the fymphyfis, Mr. Damen met with greater difficulty than before, the cartilage feeming to be harder; but as foon as the divifion was completed, the offa pubis feparated fo much, as to allow two fingers to be placed between them.

The child, which proved to be a female, was turned and delivered by the feet, as in the former operation. The circumference of this child's head was found to be fourteen inches. It died five weeks after its birth.

In the evening, after the operation, the patient was able to difcharge her urine voluntarily.

She went on well till the third day, when fhe was attacked with fymptoms of peritoneal inflammation, fuch as tenfion and pain of the belly, vomiting, and fever; to which were afterwards added, hiccup, and a fuppreffion of the milk and lochia. Thefe complaints, however, yielded to evacuations, and other fuitable remedies, and on the fixth day fhe began to recover again.

An attempt was made to apply the fame kind of belt, made of foft leather, which had been employed after the former operation; but the patient refufed to let it remain on, fo that fhe was without any bandage. This circumftance, however, did not prevent the reunion of the offa pubis, which was fo complete at the end of the third week after the operation, that the patient was able to walk.

In July 1786, fhe was examined at the Hague by Profeffor Camper and Dr. Van de Laar, who found the union of the bones perfect. She was able to ftand upright, or on either leg, without inconvenience; and had walked walked from her own houſe to Mr. Damen's, a diſtance of about a mile and a half.

On the inſide of the ſymphyſis they felt a hollow part, though very ſhallow, the cartilage, or callus, not having completely filled up the interval. Externally, no cicatrix was viſible; nor was any thing preternatural obſerved about the urethra.

She had had no incontinence of urine after the ſecond operation, and was perfectly free from any ſuch complaint at the time this examination took place. She felt no inconvenience in going to ſtool; but had a ſlight prolapſus of the vagina. Her menſes had appeared regularly.

The hernia having been neglected, filled up the pudendum on the right ſide, but was eaſily reducible.

